# Mechanical, physical and chemical characterisation of mycelium-based composites with different types of lignocellulosic substrates

**DOI:** 10.1371/journal.pone.0213954

**Published:** 2019-07-22

**Authors:** Elise Elsacker, Simon Vandelook, Joost Brancart, Eveline Peeters, Lars De Laet

**Affiliations:** 1 Architectural Engineering Research Group, Department of Architectural Engineering, Vrije Universiteit Brussel, Brussels, Belgium; 2 Research Group of Microbiology, Department of Bioengineering Sciences, Vrije Universiteit Brussel, Brussels, Belgium; 3 Research Group of Physical Chemistry and Polymer Science, Department of Materials and Chemistry, Vrije Universiteit Brussel, Brussels, Belgium; Bartin University, TURKEY

## Abstract

The current physical goods economy produces materials by extracting finite valuable resources without taking their end of the life and environmental impact into account. Mycelium-based materials offer an alternative fabrication paradigm, based on the growth of materials rather than on extraction. Agricultural residue fibres are inoculated with fungal mycelium, which form an interwoven three-dimensional filamentous network binding the feedstock into a lightweight material. The mycelium-based material is heat-killed after the growing process. In this paper, we investigate the production process, the mechanical, physical and chemical properties of mycelium-based composites made with different types of lignocellulosic reinforcement fibres combined with a white rot fungus, Trametes versicolor. This is the first study reporting the dry density, the Young’s modulus, the compressive stiffness, the stress-strain curves, the thermal conductivity, the water absorption rate and a FTIR analyse of mycelium-based composites by making use of a fully disclosed protocol with T. versicolor and five different type of fibres (hemp, flax, flax waste, softwood, straw) and fibre processings (loose, chopped, dust, pre-compressed and tow). The thermal conductivity and water absorption coefficient of the mycelium composites with flax, hemp, and straw have an overall good insulation behaviour in all the aspects compared to conventional materials such as rock wool, glass wool and extruded polystyrene. The conducted tests reveal that the mechanical performance of the mycelium-based composites depends more on the fibre processing (loose, chopped, pre-compressed, and tow), and size than on the chemical composition of the fibres. These experimental results show that mycelium-composites can fulfil the requirements of thermal insulation and have the potential to replace fosile-based composites. The methology used to evaluate the suitability and selection of organic waste-streams proved to be effective for the mycelium-material manufacturing applications.

## Introduction

The construction sector in Europe accounts for about half of all our extracted materials and energy consumption and for about a third of our water consumption and waste generation [1]. Instead of extracting raw resources that will generate future waste, biological materials can be grown based on agricultural plant-based residues. The renewable and closed-loop composites are composed of fungal biomass and lignocellulosic waste streams. The hyphae of the fungus form an interwoven three-dimensional filamentous network through the cellulose, hemicellulose and lignin rich substrate by digesting its nutrients and simultaneously binding the substrate. When reaching complete substrate colonization the organism is heat-killed above a critical temperature to render the material inert and allow the evaporation of the residual water from the material. The result is a lightweight and bio-degradable composite with a low environmental impact. This material has the potential to replace fossil-based and synthetic materials such as polyurethane and polystyrene.

The development and implementation of this material in the field of architecture and construction has hardly been investigated and characterised so far [2]. Additionally, the composite material is complex due to its wide variety of possible combinations between substrate type and fungal species. Studies on the effects between the process variables and the material behaviour are very limited [3]. Every growth parameter variation can result in changes in the material constitution and mechanical properties [3]. Furthermore, the methodologies, mycelium species and feedstocks of the published studies proceed in no standardised and comparable way [3]. Moreover, most studies use a mixture provided by Ecovative Design LLC (Green Island, NY, USA) [4–6] or Mogu (Lombardy, Italy) [7] and therefore do not fully disclose the preparation and composition of the mycelium-composites due to proprietary information, thus preventing a proper comparison or replication [5–9].

The main factors affecting the production of mycelium composites, and consequently their mechanical behaviour, are: the matrix (mycelium species [10,11]), the feedstock selection (lignocellulosic substrate [4,12]), the interaction between white rot fungi and their feedstock and last but not least the process variables during manufacturing (protocol, sterilisation, inoculation, packing, incubation, growing period and drying method). Studies have demonstrated that the mechanical properties of mycelium-composites are mostly affected by its feedstock [4,7,12]. White-rot fungi basidiomycetes are the most rapid degraders of lignin. By using phenol-oxidizing and peroxidase producing enzymes the fungi is able to depolymerise and mineralize lignin (polymer) macromolecules [13]. The chemical structure of lignin therefore changes during the enzymatic oxidation with laccase and peroxidase [14,15]. During this oxidation process, lignin-based radicals can be cross-linked and subsequently form an adhesive between the fibres [13]. *T*. *versicolor* is known to be a non-selective white-rot, it removes lignin and structural carbohydrates (hemicellulose, cellulose) at a similar rate [16,17].

The purpose of this research is to further investigate the effects of different types of agricultural by-product fibres and different fibre processing on the material properties. This study aims to determine the influence of the composition of fibres on growth performances of *Trametes versicolor* and establish a methodology to evaluate the suitability of those fibres for the production of mycelium-based building materials.

## Materials and methods

### Natural fibres

**Fig 1:**
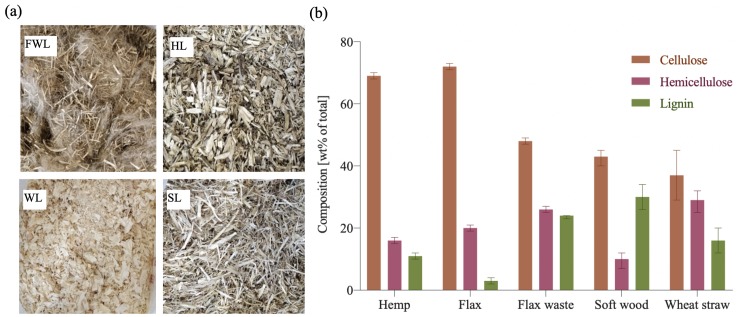
Composition of fibre types. (a) loose flax waste (FWL), loose hemp (HL), loose wood (WL), loose straw (SL). (b) Composition of agricultural by-products. Compiled from literature [18–20].

The selected natural fibres (Fig 1, Table 1) were: flax, flax dust, flax long treated fibres, flax long untreated fibres, flax waste, wheat straw dust and wheat straw, and were obtained from Jopack bvba (Rumbeke, Belgium) (Fig 1a). The hemp fibres and pine softwood shavings were purchased from Aniserco S.A (Groot-Bijgaarden, Belgium). The treated fibres were very similar in appearance to the untreated fibres but were slightly softer to the touch and ready for spinning to yarn, while the untreated fibres had more residual small fibres. The loose fibres (hurds) were the inner part of the stem which was a left over product when the bark fibre was removed. The chopped fibres were processed in a blender (Emerio BL-108862) during 10 minutes to reach a fibres size smaller than 5 mm.

**Table 1:** Summary of the natural fibre types, processing, type of mould and type of test, with the corresponding labels.

Fibre group	Source	Type	Processing	Particle size (mm)	Label	Type of mould	Type of test
Fibre crops	Bast	Hemp	Loose (hurds)	5–25	HL	M1 (75 x 37,5 mm)	Compression
			Chopped	< 5	HC	M1 (75 x 37,5 mm)	Compression
						M3 (63 x 200 mm)	Thermal conductivity
						M4 (20 x 100 mm)	Absorption of water
			Pre-compressed loose (hurds)	5–25	PHL	M2 (75 x 100 mm)	Compression
		Flax	Loose (hurds)	5–25	FL	M1 (75 x 37,5 mm)	Compression
			Chopped	< 5	FC	M1 (75 x 37,5 mm)	Compression
						M3 (36 x 200 mm)	Thermal conductivity
						M4 (20 x 100 mm)	Absorption of water
			Treated tow	100	FTT	M1 (75 x 37,5 mm)	Compression
			Untreated tow	100	FUT	M1 (75 x 37,5 mm)	Compression
			Dust	0,5–1	FD	M1 (75 x 37,5 mm)	Compression
			Pre-compressed losse (hurds)	5–25	PFL	M2 (75 x 100 mm)	Compression
		Flax waste	Loose (hurds)	5–25	FWL	M1 (75 x 37,5 mm)	Compression
			Pre-compressed loose (hurds)	5–25	PFWL	M5 (75 x 60 mm)	Compression
	Wood	Pine soft wood	Loose (shavings)	5–25	WL	M1 (75 x 37,5 mm)	Compression
Agricultural residues	Cereal plants	Wheat straw	Loose (hurds)	5–25	SL	M1 (75 x 37,5 mm)	Compression
			Chopped	< 5	SC	M3 (63 x 200 mm)	Thermal conductivity
						M4 (20 x 100 mm)	Absorption of water
			Dust	0,5–1	SD	M1 (75 x 37,5 mm)	Compression

### Strain and culture conditions

The mycelium spawn *Trametes versicolor* (M9912-5LSR-2 O447A) was purchased from Mycelia bvba (Nevele, Belgium). It was conserved on a grain mixture at 4°C in a breathing Microsac bag (Sac O2 nv, Nevele, Belgium).

### Sample preparation

#### Fibre preparation

The fibres intended to chop were first soaked in water for 24 hours, then rinsed abundantly, blended during 10 minutes with fresh water. The fibres were sieved with a 5mm strainer, squeezed manually, spread on a plate and dried at 30 °C for 24 hours. The dried chopped fibres were then transferred in an autoclavable micro box, 185 x185 x 78mm (obtained from Sac O2 nv, Nevele, Belgium). The loose, tow and dust fibres were not processed and were directly transferred to a micro box. The fibres were autoclaved for 20 minutes at 121°C. The boxes were left to cool down for 24 hours.

#### Types of moulds

The moulds for the compression test were fabricated from a hollow PVC tube that were composed of two demountable parts. A diameter to height ratio of 2:1 was selected for compressive tests; the test samples have a diameter of 75 mm and a height of 37,5 mm (M1). For pre-compressed samples, moulds of diameter 75 mm and height 100 mm (M2) were used. PVC moulds fabricated for the thermal conductivity tests were based on ASTM D5334 [21], NBN EN 1609 [22] and NBN EN 12667 [23] and had a diameter of 63 mm and height of 200 mm (M3). To define the rate of absorption of water by partial immersion the norms NBN EN ISO 15148 [24] and ASTM C 1585–04 [25] were applied, and PVC moulds of 20 mm in diameter and 100 mm height were used (M4).

### Composite fabrication

#### Growth

**Fig 2:**
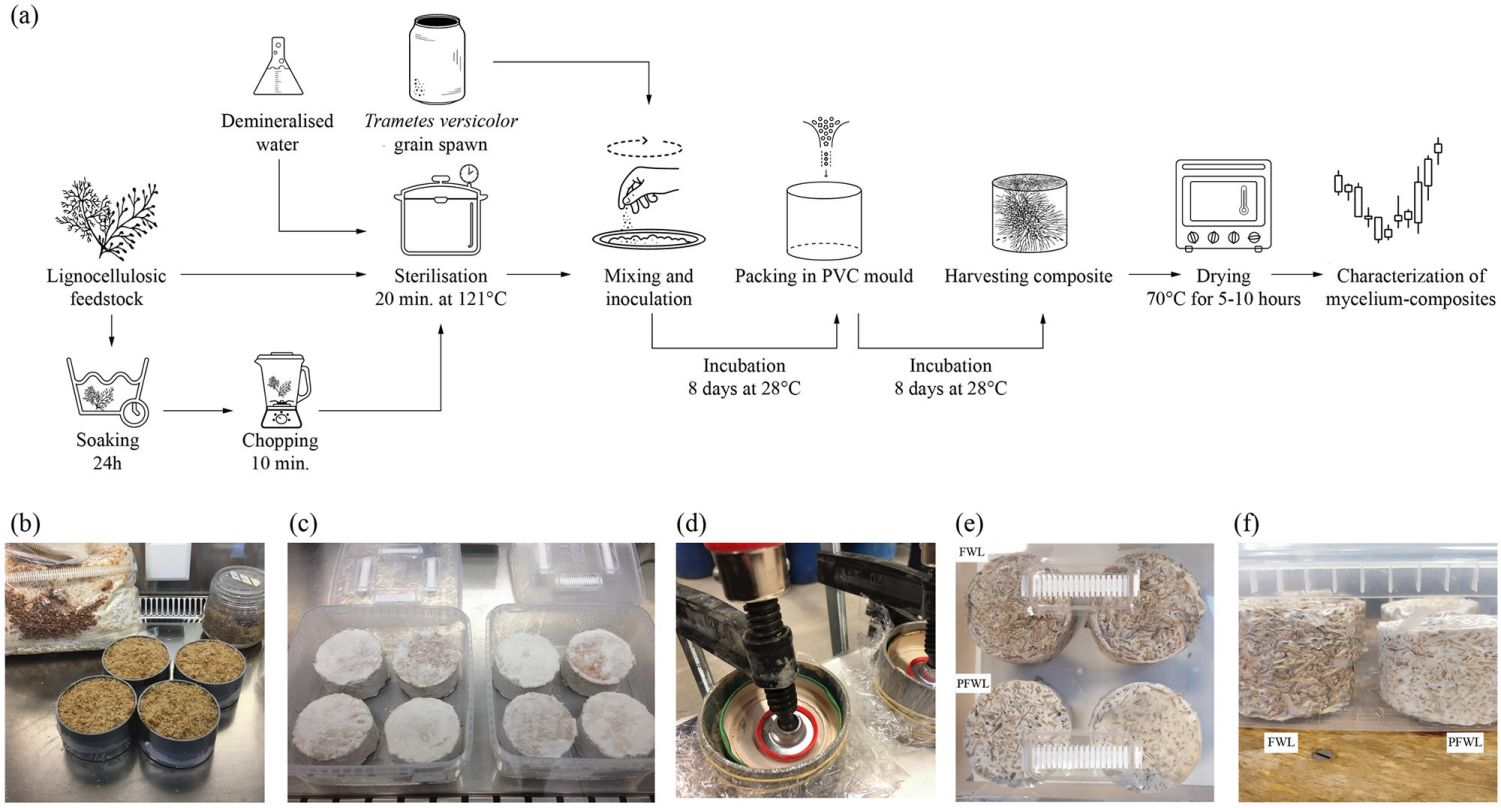
Fabrication process of mycelium-based composites. (a) Process flow chart showing the applied fabrication method of mycelium-based composites. (b) Preparation of samples in moulds for the compression test in the laminar flow. (c) Fully grown samples after 16 days. (d) Pre-compressed PHL, PFL, PFWL samples. (e-f) Demoulded FWL (above) and PFWL (under) samples after 8 days at 28°C, the second growth phase is initiated in a sterile microbox with filters.

The 20% wt (weight percentage) of fibres, 70% wt of sterile demineralised H_2_O and 10% wt of mycelium spawn were mixed together and put in the PVC moulds (Fig 2a). The moulds were filled by layers, while compressing each layer with a spoon to obtain a compact and dense sample (Fig 2b) and covered with a transparent perforated cellophane foil. For all groups (Table 2), three replicates were made. Three extra undried samples (HL, FWL, FL) were selected for visual inspection of the cross-sections (S1 Fig). Those were cut open in the middle with a knife. In parallel to the fabrication of the composite in moulds, samples were also grown in squared transparent petri-dishes (120 x 120 x 17mm) to follow the growth by visual inspection day by day (S2 Fig).

**Table 2:** Experimental design of composite fabrication.

Label	Weight before drying [g]	Weight after drying [g]	Dry density [kg/m3]	Initial moisture content [%]	Diameter [mm]	Height [mm]	Average shrinkage [%]
HL (C)—a	38,5 ± 2	10,5 ± 0,5	88,6 ± 1,4^h-k,o^	72,8 ± 1,8^n,s^	69,7 ± 0,5	30,9 ± 1,4	9% ± 2,3^d,i-k,o,p^
HC (C)—b	60,2 ± 6,1	12,0 ± 0,8	97,4 ± 3,2^j,o^	80,1 ± 0,7^c,p,r^	70,4 ± 0,9	31,7 ± 0,3	11% ± 1,0^d,k,o^
HC (Tc)—c	128,1 ± 0,0	43,1 ± 0,0	98,9 ± 0,0^f,i,j,o^	66,4 ± 0,0^f,g,i,k-o,s^	54,6 ± 0,0	185,7 ± 0,0	10% ± 0,0^d,j,k,o^
HC (Aw)—d	6,6 ± 0,0	1,6 ± 0,0	98,4 ± 4,6^f,i,j,o^	75,1 ± 1,7	15,4 ± 0,7	89,4 ± 0,4	17% ± 1,6^e,f,h^
PHL (C)—e	84,2 ± 0,1	24,6 ±1,4	72,1 ± 0,2^h-k,o,s^	70,8 ± 1,6^g,n,s^	72,1 ± 1,1	83,5 ± 7,0	10% ± 2,8^j,k,o^
FL (C)—f	34,4 ± 2,2	7,0 ± 1,3	59,8 ± 11,7^h-k,o,s^	79,5 ± 4,6^p,r^	70,7 ± 0,3	30,1 ± 0,2	10% ± 0,5^j,k,o^
FC (C)–g	112,5 ± 6,3	21,2 ±1,2	65,8 ± 4,2^h-k,o,s^	81,2 ± 0,0^j,p,r^	74,0 ± 1,2	75,1 ± 5,2	13% ± 1,8^k^
FC (Tc)—h	225,2 ± 0,0	56,7 ± 0,0	134,7 ± 0^j,m,n,p,r^	74,8 ± 0,0	53,8 ± 0,0	185,3 ± 0,0	11% ± 0,0^k,o^
FC (Aw)—i	12,1 ± 0,1	2,5 ± 0,1	137,5 ± 2,9^j,m,n,p,r^	61,7 ± 0,4^p,r^	16,1 ± 0,4	90,0 ± 0,5	15% ± 0,7^k^
FTT (C)—j	62,8 ± 2,6	18,6 ± 4,0	187,3 ± 35,6^k-n,p-s^	70,4 ± 5,1^s^	70,1 ± 0,3	25,7 ± 0,4	17% ± 0,7
FUT (C)—k	59,3 ± 18,7	11,6 ± 1,3	132,2 ±10,1^m,p,r^	80,4 ± 3,5^p,r^	68,0 ±0,8	24,2 ± 1,4	20% ± 1,8^m,o,p-s^
FD (C)—l	n/a *	n/a *	n/a *	n/a *	n/a *	n/a *	n/a *
PFL (C)—m	92,7 ± 3,5	22,1 ± 0,7	68,5 ± 2,2^o,s^	76,1 ± 0,6	74,0 ± 1,2	75,1 ± 5,2	13% ± 1,8
FWL (C)—n	53,6 ± 3,9	9,6 ± 1,2	96,5 ± 13,5^o^	82,0 ± 2,6^p,r^	67,3 ± 1,3	28,1 ± 1,5	15% ± 1,3
PFWL (C)—o	111,4 ± 15,6	27,0 ± 1,1	159,5 ± 6,6^p-s^	75,8 ± 3,6	73,6 ± 0,0	39,8 ± 0,0	18% ± 0,0
WL (C)—p	29,3 ± 3,7	8,8 ± 1,0	87,4 ± 5,2	70,1 ± 1,2^s^	67,5 ± 1,8	28,1 ± 0,2	15% ± 1,3
SL (C)—q	n/a *	n/a *	n/a *	n/a *	n/a *	n/a *	n/a *
SC (Tc)—r	121,7 ± 0,1	37,9 ± 0,0	94,4 ± 0,0	68,9 ± 0,0^s^	53,6 ± 0,0	177,7 ± 0,0	13% ± 1,9
SC (Aw)—s	13,45 ± 0,5	2,3 ± 0,3	122,1 ± 10,9	83,2 ± 2,0	15,7 ± 0,4	94,8 ± 0,4	13% ± 0,9
SD (C)—t	n/a *	n/a *	n/a *	n/a *	n/a *	n/a *	n/a *

Type of fibre: H: Hemp, F: Flax, FW: Flax waste, W: Wood, S: Straw. Fibre processing: L: Loose, C: Chopped, P: Pre-compressed, TT: Treated Tow, UT: Untreated Tow, D: Dust. Type of tests: (C): compression, (Tc): Thermal conductivity, (Aw): Absorption of water. The standard deviation is performed with triplicate specimens (mean ± one standard deviation). Lettres indicate significant differences based on Tukey’s family error rate at p≤0.05 for sample-specific ANOVA, for the dry density, moisture content and shrinkage.

* Specimens failed to grow.

#### Incubation

The samples (Fig 2b) were incubated in a micro box with a depth-filtration system that allows for air flow at 28°C for 8 days. After 8 days, the samples were demoulded in the laminar flow and incubated in a micro box for another minimum of 8 days without mould (Fig 2c) in order to achieve an homogenous colonisation on the sides that were in contact with the PVC mould.

The PHL, PFL, PFWL samples were pre-compressed after 8 days. The samples were placed on a base plate that was fixed on a table. Every sample was provided with a cover as surface barrier between the sample and the screw clamp to distribute the compression force over the top surface. Two screw clamps were required for every sample, one to compress and one to keep the demountable parts of the mould closed on the sides (Fig 2d). The samples were compressed from an initial height of 100 mm to 80 mm during the second growth phase of 8 days. The samples were demoulded in the laminar flow and incubated in a microbox for another 3 days without mould (Fig 2d–2f).

#### Drying process

All samples were dried in a convection oven at a temperature of 70 °C for 5 to 10 hours, until their weight stabilised [26]. Every sample was weighted before and after drying, along with the diameter and height. Shrinkage percentage was calculated by subtracting the dry volume weigth from the wet volume weigth and dividing this shrinkage with the wet volume. Averages of all replicates are presented in Table 2. For two extra specimens (loose hemp and flax) the weight loss was measured every 60 min during 6 hours to determine the humidity decrease over time.

### Composite characterization

#### Dry density

The density was calculated following ISO 9427:2003 [27], by taking the ratio of the oven-dry mass over the volume (Table 2).

#### Moisture content

The moisture content was calculated following ISO 16979:2003 [28] with the formula:

$$\text{M}=\frac{\left(W_{w}-W_{d}\right)*100}{W_{d}}\left[\%\right]$$

where:

M = moisture content [%], w_w_ = wet weight [g], w_d_ = oven dry weight [g] (Table 2).

#### Mechanical testing in compression

Compressive stiffness was determined following ASTM D3501 [29] on an Instron 5900R load bench with a 100 kN capacity and a 10 kN load cell at ambient conditions (25°C and ~ 50% RH). The 10 kN load cell was used to have the most accurate results since low ultimate loads values were expected. The tests were displacement controlled with a rate of 5 mm/min. The contact surface was not perfect due to the rough surfaces of the samples. The test was stopped when a fixed strain was reached in the specimen, varying between 70% and 80%. The load-displacement curve was converted to a stress-strain curve, using the following formulas to calculate the compressive stress σ and the strain ε:

$$\sigma =\frac{F}{A}\left[\text{MPa}\right]$$

and

$$\varepsilon =\frac{\Delta L}{Lo}\left[-\right],$$

where:

F = compressive force [N], A = original cross section of the specimen [mm^2^], ΔL = displacement of the loading surfaces [mm] and L_o_ = original height of the test piece [mm].

#### Thermal conductivity

The transient method (non-steady conditions needed) was used to measure the thermal conductivity according to ASTM D 5334–00 [21]. The thermal needle probe (TNP) (produced by Huksefluxand with commercial reference TP02), complies with the standard. A first guide hole (2mm) was drilled in the centre of the cylindric sample with the help of an extra needle with a smaller diameter than the TNP. There is one deviation from the ASTM standard: a different electrical current (S1 Table, S3 Fig) in the circuit was applied. When a high current is applied, the temperature increases faster, resulting in a lower thermal condictivity. The fast rise in temperature is not recommended by the standard due to possible errors while reading. In order to have reliable results, an average was taken between high currents and low currents. The thermal conductivity is determined by:

$$\lambda =\frac{Q}{4\pi (T_{2}-T_{1})}*\text{ln}\;\left(\frac{t2}{t1}\right)$$

where:

Q = constant current [W/m], T_1, 2…_ = initial and final temperature of the linear portion [°C], t_1, 2…_ = initial and final time of the linear portion [s]

#### Rate of water absorption

The method for Measurement of Rate of Absorption of Water by Hydraulic-Cement Concretes ASTM C 1585 [25] with partially submerged samples was applied to the mycelium composites. The tests were conducted at room temperature (20°C). The water absorption was determined from the weight difference in relation to the initial weight. The formula to determine the water absorption is:

$$m=\frac{M_{t}}{a*d},$$

where:

m = water absorption [mm], M_t_ = changed weight of the specimen [g], a = the exposed cross-section area [mm^2^], d = the density of the water [g/mm^3^].

#### Chemical characterization

For the Fourier Transform Infrared Spectroscopy all IR spectra were acquired on a Nicolet 6700 FT-IR spectrometer from Thermo Fischer Scientific. The FTIR instrument was equipped with an IR source, DGTS KBr detector and KBr beamsplitters and windows. FTIR spectra were recorded in single bounce Attenuated Total Refractance (ATR) mode using the Smart iTR accessory, equipped with a diamond plate (42° angle of incidence). The spectra were recorded with automatic atmospheric correction for the background.

All samples were measured at a spectral resolution of 4 cm^-1^ with 64 scans per sample. Four samples of each type were measured to ensure the reproducibility of obtained spectra [12]. Peak height, area and subtraction was measured using Spectragryph v1.2.8 software. The spectra were cut between 4000 and 700 cm^-1^ bands, then the baseline was constructed by connecting the lowest data point on either side of the peak, and finally the peaks were normalised by surface area. The maximum absorbance intensities for lignin associated bands obtained from the software were divided against carbohydrate reference peaks in Excel.

### Statistical analysis

The dry weight, moisture content, density, shrinkage, water absorption, thermal conductivity, and Young’s modulus were statistically analysed in Microsoft Excel and graphed with GraphPad Prism (version 8.1.2). Data were checked for normality (p≥0.05) using Sharpiro-Wilk test. An one-way analysis of variance (ANOVA) was used for normal data and significant differences were considered at p ≤ 0.05. The multiple comparisons test for normal data was generated based on Tukey’s family error rate. For non-parametric data, the Kruskal-Wallis test was conducted and significant differences were considered at p ≤ 0.05. The Dunn’s multiple comparison test was used for the non-parametric data.

## Results and discussion

### Sample description and growth examination

Variations in the natural fibres were based on two different aspects: the fibre type and the fibre condition (loose, chopped, dust, pre-compressed and tow (Fig 1 and Table 1). The chosen substrates had a chemical composition (Fig 1b) which was bio-compatible with the selected white-rot fungus [30]. Since no previous study analysed the bio-compatibility of the fibre processing, a visually inspection of the growth performances was conducted as a first stage in the development of mycelium-composites to determine the compatibility of the fungi with the selected fibres. The growth evolution of a representative selection of the samples is presented in S2 Fig. The samples with flax dust (FD), pine softwood (W) and straw (S) poorly grew after 10 days. A slow growth and systematic contamination for all flax dust (FD) and straw dust (SD) samples was observed. Therefore, no further test were conducted with this type of fibre. We observed a dense white fungal biomass layer formed over the hemp (HL), flaw (FL), flax waste (FWL) and flaw tow (FTT and FUT) specimen. To analyse the inner growth of the specimens, undried samples of hemp, flax and flax waste were cut open (S1 Fig). Despite the spawn that was mixed throughout the whole substrate during inoculation, poor growth was observed inside the samples. A possible explanation for the low colonisation inside the sample might be related to the growth conditions: absence of light and air, and the accumulation of heat produced by mycelium during growth [31]. The outer surface grew, whereas internal growth did not continue.

**Fig 3:**
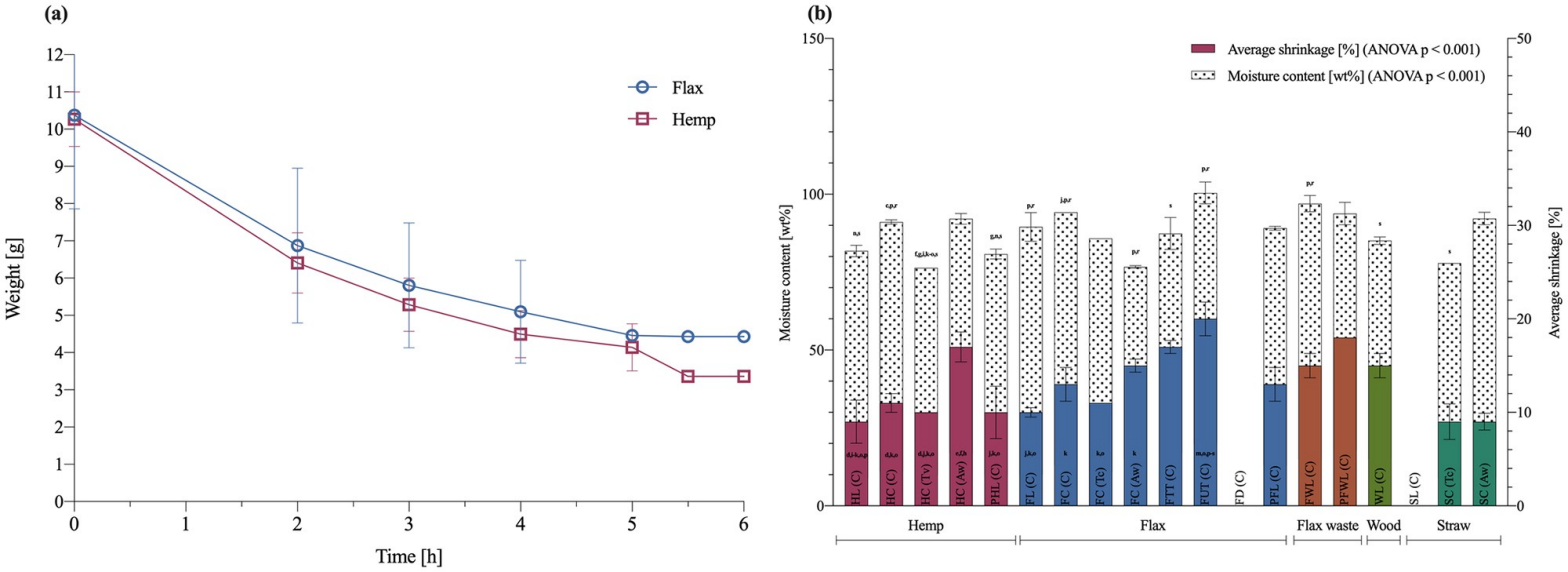
Drying curve, shrinkage percentage and moisture content after drying mycelium-based materials. (a) Drying curve for the loose hemp and flax samples at a temperature of 70°C. (b) Average shrinkage percentage of the diameter and height (solid), and moisture content (pattern) per fibre type and condition. The standard deviation is performed with triplicate specimens (mean ± one standard deviation). Lettres indicate significant differences based on Tukey’s family error rate at p≤0.05 for sample-specific ANOVA, for the moisture content and shrinkage.

The initial weight, the dry weitght, dry density, initial moisture content and dimentional stability of all samples is shown in Table 2. By measuring the weight of two selected samples (loose hemp and flax) during drying, their humidity decrease was determined over time (Fig 3a). The highest decrease in weight took place during the first two hours of drying, followed by a more or less constant behaviour for the third hour, after which weight decreased again until stability was reached after 5h. The weight of the loose hemp samples decreased more (69%) compared to the loose flax samples (60%). After the drying process the volume of all samples decreased due to the evaporation of the water (Fig 3b). There was no noticeable difference in shrinkage between the fibre processing: loose, chopped, pre-compressed, and tow. The dry density varied depending on the used substrate for all samples (Table 2), and was the lowest for loose flax with 59,77 kg/m^3^ and the highest for treated tow flax with 187,29 kg/m^3^.

### Mechanical behaviour in compression

As no standard testing procedure exists for mycelium-based composites, different geometries of moulds were tested in a first iteration based on literature [8,31–33]. Eventually a diameter to height ratio of 2:1 was selected for compressive tests. The large contact area with the loading bench resulted in a more distributed stress induction, while the limited height prevented failure by buckling.

**Fig 4:**
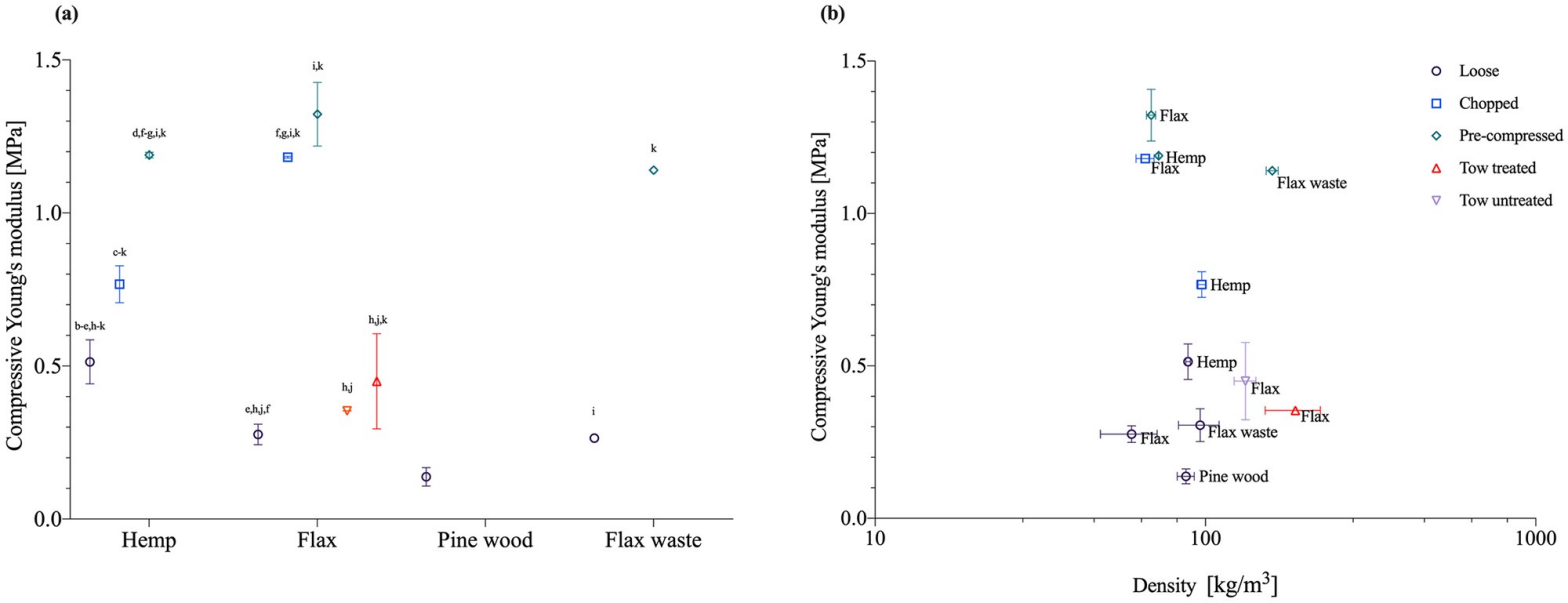
Compressive Young’s modulus of mycelium-based composites. (a) Modulus of elasticity of mycelium-composites during uniaxial compression with different types of fibres (hemp, flax, wood and flax waste) and different fibre processing (loose, chopped, tow treated, tow undtreated, pre-compressed). (b) Overview of the Young’s modulus [MPa] versus density [kg/m^3^]. The standard deviation is performed with triplicate specimens (mean ± one standard deviation). Lettres indicate (lable in Table 2) significant differences based on Tukey’s family error rate at p≤0.05 for sample-specific ANOVA.

The modulus of elasticity shown in Fig 4a and 4b was measured to reveal the resistance of the material in compression and thus its stiffness. The mechanical compressive stiffness was obtained from the slope of stress-strain curve (S4 Fig) with the tangent modulus. The values were in correspondence with the preceding growth observations: the samples with a dense white fungal biomass homogenous colonisation resulted in the highest compressive stiffness. Within the group of loose fibres the combination of mycelium and hemp achieved the highest compressive stiffness (0,51 MPa), followed by flax waste (0,31 MPa). The lowest stiffness is achieved by the wood samples (0,14 MPa). We can also observe an increase in compressive stiffness for chopped hemp (0,77 MPa) and chopped flax (1,18 MPa) compared to loose hemp (0,51 MPa) and loose flax (0,28 MPa), indicating that the fibre condition and smaller fibre size influences the compressive stiffness. The compressive stiffness is considerably larger for chopped flax, compared to any other fibre type and condition. Chopped fibre substrates result in mycelium composites with slightly higher densities compared to the samples with a loose substrate. For loose hemp and chopped hemp, the density remains approximately equal, which might explain the small change in stiffness. For chopped flax fibres the increased composite’s density influences its stiffness. Flax fibres have the highest stiffness in a chopped condition and the lowest one in a loose condition, whereas the results for hemp-based substrates are less spread. The compressive strength for flax treated tow and flax untreated tow resulted in 0,35 MPa and 0,45MPa, which can be related to the higher density of the composite.

Additional pre-compressed samples of loose hemp, flax and flax waste were grown to optimize the compressive stiffness. Pre-compression of the samples took place during manufacturing, before demoulding and before drying. The aim was to improve the compactness and thus compressive properties of the composites. The action of pre-compressing samples influences their mechanical response in terms of compressive stiffness, resulting in a Young’s modulus of 1,19 MPa for pre-compressed loose hemp, 1,32 MPa for pre-compressed loose flax, and 1,14 MPa for pre-compressed loose flax waste.

### Thermal conductivity

To know whether the mycelium-composites could be used as an alternative biological insulation material, the thermal conductivity was measured for chopped hemp, flax and straw fibres (S1 Table, S3 Fig). Since the standard prescribes a specific particle size, the tests were only conducted on the chopped fibre condition.

The value of the thermal conductivity (Table 3) for flax waste measures 0.0578 W/ (m*K), which is larger than hemp and straw, respectively 0.0404 W/ (m*K) and 0.0419 W/ (m*K). The larger thermal conductivity for flax can be attributed to the higher density of the samples (134,71 kg/m^3^) (Table 2). The values of the thermal conductivity for hemp and straw ranged within the same values as conventional insulating materials (Table 3) such as rock wool, glass fibres, sheep wool, cork [34–36]. The results of this study also showed better thermal insulation properties of the investigated samples compared to recent research on mycelium-based biofoams [26] with values between 0.05–0.07 W/ (m*K), and [11] with values between 0.078 and 0.081 W/ (m*K). This can be attributed to a different production protocol of the samples. In the first study [26], the species *I*. *lacteus* was grown on sawdust pulp of Alaska birch. The composites had a density between 180 and 380 kg/m^3^. In the second study [11], the species *Oxyporus latermarginatus*, *Megasporoporia minor* and *Ganoderma resinaceum* were inoculated in wheat straw, producing composites with densities between 51 and 62 kg/m^3^. The results proved that mycelium composites can become an alternative biological insulation material whereby the properties are influenced by the fabrication variables.

**Table 3:** Summary of thermal conductivity, density of mycelium-based composites and conventional insulation materials.

Insulating Material	Thermal conductivity [W/(m*K)]	Density [kg/m^3^]	Source
Mycelium-flax composite—a	0.0578 ± 0,002^bc^	135	This work
Mycelium-hemp composite—b	0.0404 ± 0,001^a^	99	This work
Mycelium-straw composite—c	0.0419 ± 0,0002^b^	94	This work
Rock wool	0.044	470–2250	[34]
Glass wool	0.033–0.045	13–100	[35]
Extruded polystyrene	0.025–0.035	18–50	[35]
Kenaf	0.034–0.043	30–180	[36]
Sheep wool plates	0.038–0.054	10–25	[36]

The standard deviation is performed with triplicate specimens (mean ± one standard deviation). Lettres indicate significant differences based on Tukey’s family error rate at p≤0.05 for sample-specific ANOVA.

### Water absorption rate

**Fig 5:**
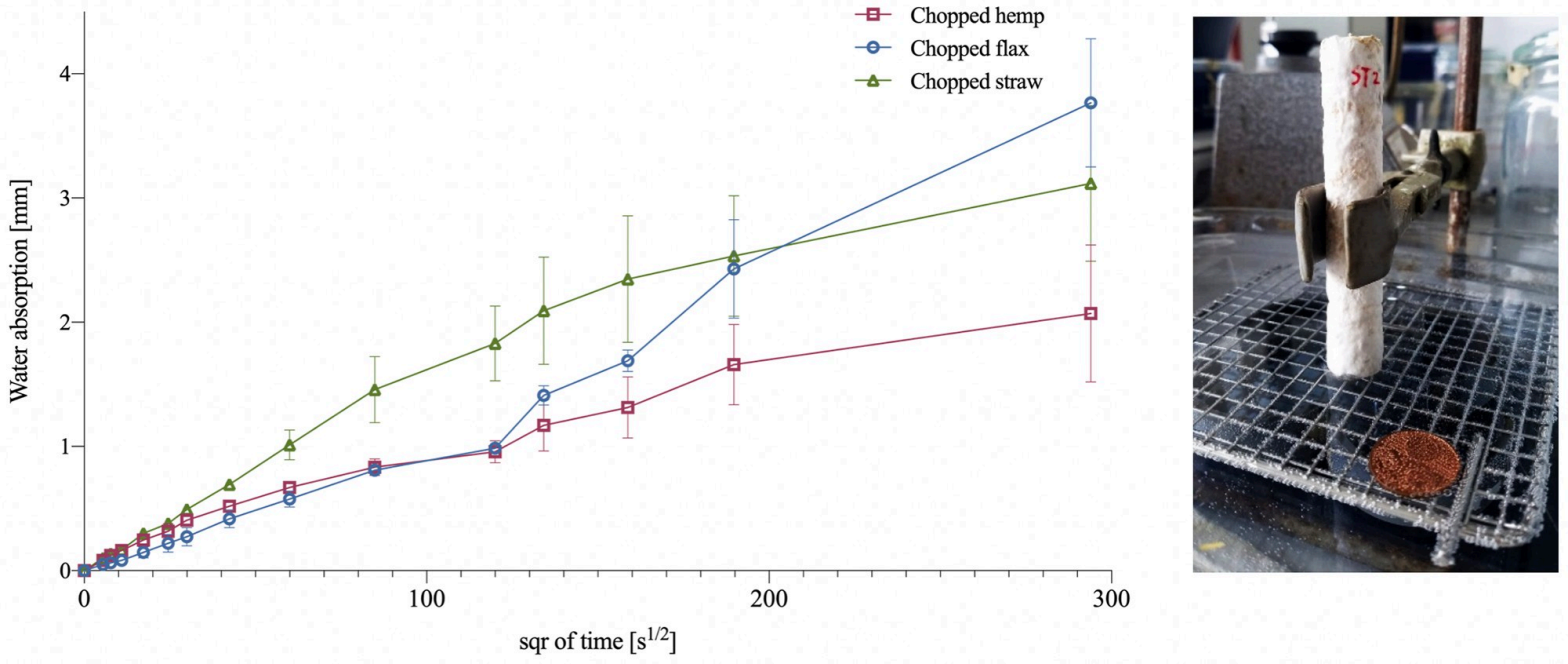
Plot of the water absorption of chopped flax (FC), hemp (HC) and straw (SC). The standard deviation is performed with triplicate specimens (mean ± one standard deviation). No significant differences were found based on Tukey’s family error rate at p≤0.05 for sample-specific ANOVA.

The water absorption rate is an important factor for the application of mycelium-composite as indoor particle boards or insulation elements, as it will determine the durability in time of the material. Fig 5 shows the water absorption in relation to the time the specimen has been submerged. During the first 30 min. the samples had an initial similar absorption rate (flax: 0,01 mm/s^1/2^, hemp: 0,012 mm/s^1/2^ and straw: 0,016 mm/s^1/2^). Only after 30 to 40 minutes, a linear trend appeared for chopped straw and hemp, which respectively lasted for 7 to 10 hours, after which the water absorption rate reduced. Consequently, the samples containing straw and hemp absorbed water in a shorter period and reached their limit of absorption faster than chopped flax. During the first 5 hours of the test the water absorption rate of flax and hemp are similar. After 5 hours flax started to absorb more water than HC. For the flax samples a non-linear behaviour appeared during the 24 hours of the test and the water absorption rate increased after 10 hours. Previously reported values by Ziegler et al., (2016) also displayed a non-linear nature of the water absorption curve [4] with woven hemp mats. This behaviour was assigned to the hydrophobic nature of mycelium (“hydorphobines” [37,38]) and the hydrophilic nature of the fibres [4]. The density of the samples can influence the diffusion coefficient of water. The low density of hemp might have affected the internal water transport. Moreover, as previously discussed the growth on hemp samples resulted in a denser outer hydrophobic mycelial layer, compared to flax and straw, explaining the lower water absorption rate of hemp.

**Fig 6:**
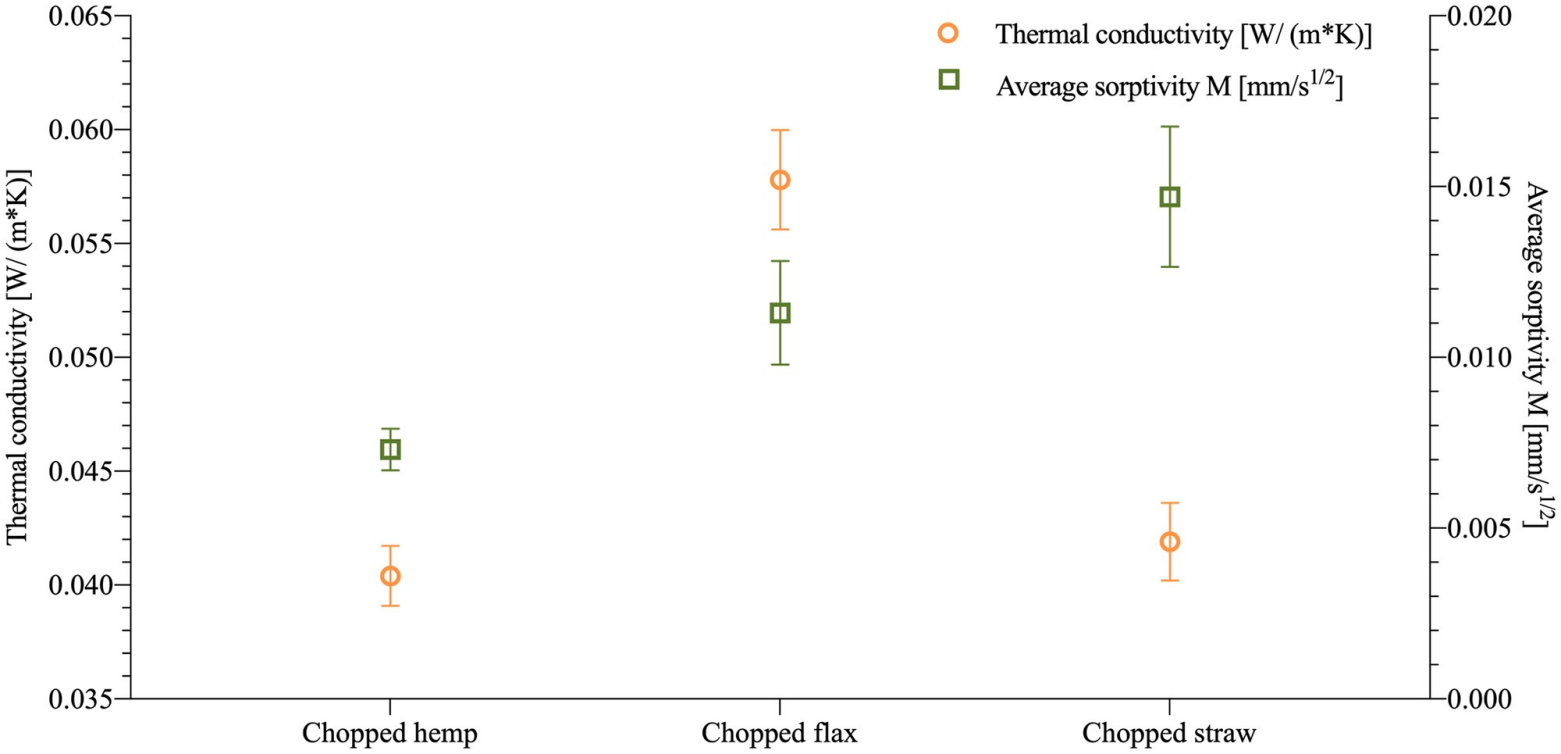
Comparison of the thermal and water absorption properties of different types of fibres (hemp, flax an straw).

The velocity of chopped hemp (0,0073 mm/s^1/2^) samples to absorb water is lower than chopped flax (0,0113 mm/s^1/2^) and straw (0,0147 mm/s^1/2^) (Table 4). After 24 hours flax composites had absorbed the highest amount of water (30,28%) compared to hemp (24,45%) and straw (26,78%). A similar behaviour was observed with hemp, the materials were not yet in a steady state after 24 hours and had the lowest absorption coefficient. Mycelium-composites made with chopped hemp are considered as interesting for construction purposes because of their low water absorption coefficient, and low absorption rate in 24 hours (Fig 6). All mycelium-composites have lower coefficients than clay bricks (0,019 mm/s^1/2^), mortar (0,011 mm/s^1/2^), glass fibres (0,049 mm/s^1/2^). In other research, the water absorption properties after 24 hours were reported to be much higher, between 180% and 350% [4,5]. This might be explained by the fact that the samples of both studies were heat or coldpressed resulting in a shattered, damaged outer mycelial layer, whereas the samples of this study were grown with a fully grown and dense mycelial outer layer.

**Table 4:** Summary of the water absorption rate of chopped flax (FC), hemp (HC), straw (SC).

Fibre type	Average water absorption rate M [mm/s^1/2^]	Water absorbed after 24 hours [%]	Density [kg/m^3^]
Chopped hemp	0.0073 ± 0.0006	24.45	98,4 ± 4,6
Chopped flax	0.0113 ± 0.002	30.28	137,5 ± 2,9
Chopped straw	0.0147 ± 0.002	26.78	122,1 ± 10,9

The standard deviation is performed with triplicate specimens (mean ± one standard deviation).

### Chemical characterization

Since white-rot decay influences the chemistry of the fibres, a FTIR analysis was performed to understand the chemical composition of different feedstock by analysing the appearance and disappearance of bands. This is the first reported FTIR analysis on mycelium-composites. Spectra of all decayed and undecayed fibres presented in Table 1 were recorded, but in this paper we only compare flax and hemp fibres, as previous discussed results for those fibres proved the most interesting advantages for construction applications. The peak position and intensities of the bands with respect to the baseline are listed in Table 5.

**Table 5:** Peak assignment for the FTIR characterization of T. versicolor grown on flax.

Wave number (cm^-1^)	Peak (cm^-1^)	Assignment	Reference
3600–3000	3280	O-H stretching hydrogen bonds	[12,39]
3000–2840		C-H stretching in methyl and methylene groups	[17,40]
2980–2835	2922, 2850	CH_2_, CH_2_OH in cellulose	
2985–2947	2960	CH_2_ asymmetric stretching	[40]
2940–2840	2850	CH_2_ symmetric stretching	
1770–1710	1733	C = O stretching in xylans (hemicellulose)	[17,39]
	1633	Absorbed O-H associated with lignin or cellulose	[17,40]
1560–1520	1551	C = C stretching of aromatic ring (syringyl) in lignin	[17,40]
1520–1500	1510	C = C stretcing of aromatic ring (guaiacyl) in lignin	[17,39,40]
1470–1410		C-H deformation in methyl groups	[41]
	1456, 1419	C-H deformation in lignin and carbohydrates, CH_2_ bending	[12,17,39,40]
1375–1365	1370	CH bending in cellulose and hemicellulose, chitin	[12,17,39,40]
1365–1335		OH plane deformation vibration	[40]
	1317	CH_2_ wagging in cellulose	[17,39,40]
1282–1277		CH deformation in cellulose	[40]
	1262	Guaiacyl ring breathing, C-O linkage in guaiacyl aromic methoxyl groups	[17,40]
	1240	Sycingyl ring, C-O stretching in lignin and xylan, Nuclei acide	[12,17,39]
1235–1225		OH plane deformation	[40]
1205–1200		OH plane deformation in cellulose, C-C stretching, C-O stretching, C-H deformation,	[12,40]
1162–1125	1143	C-O-C vibration in cellulose and hemicellulose	[17,39,40]
		Aromatic C-H in-plane deformation	[40]
	1105	Aromatic C-H in-plane deformation, C = O stretch	[40]
1060–1015		C-O valence vibration from C3-O3H	[40]
1047–1004		C-O stretching in cellulose	[17,39,40]
	1031	C-C stretching	[12]
	896	Anomere C-group, Glucan ß-anomer C-H bending, C-H deformation in cellulose	[12,17,39,40]

**Fig 7:**
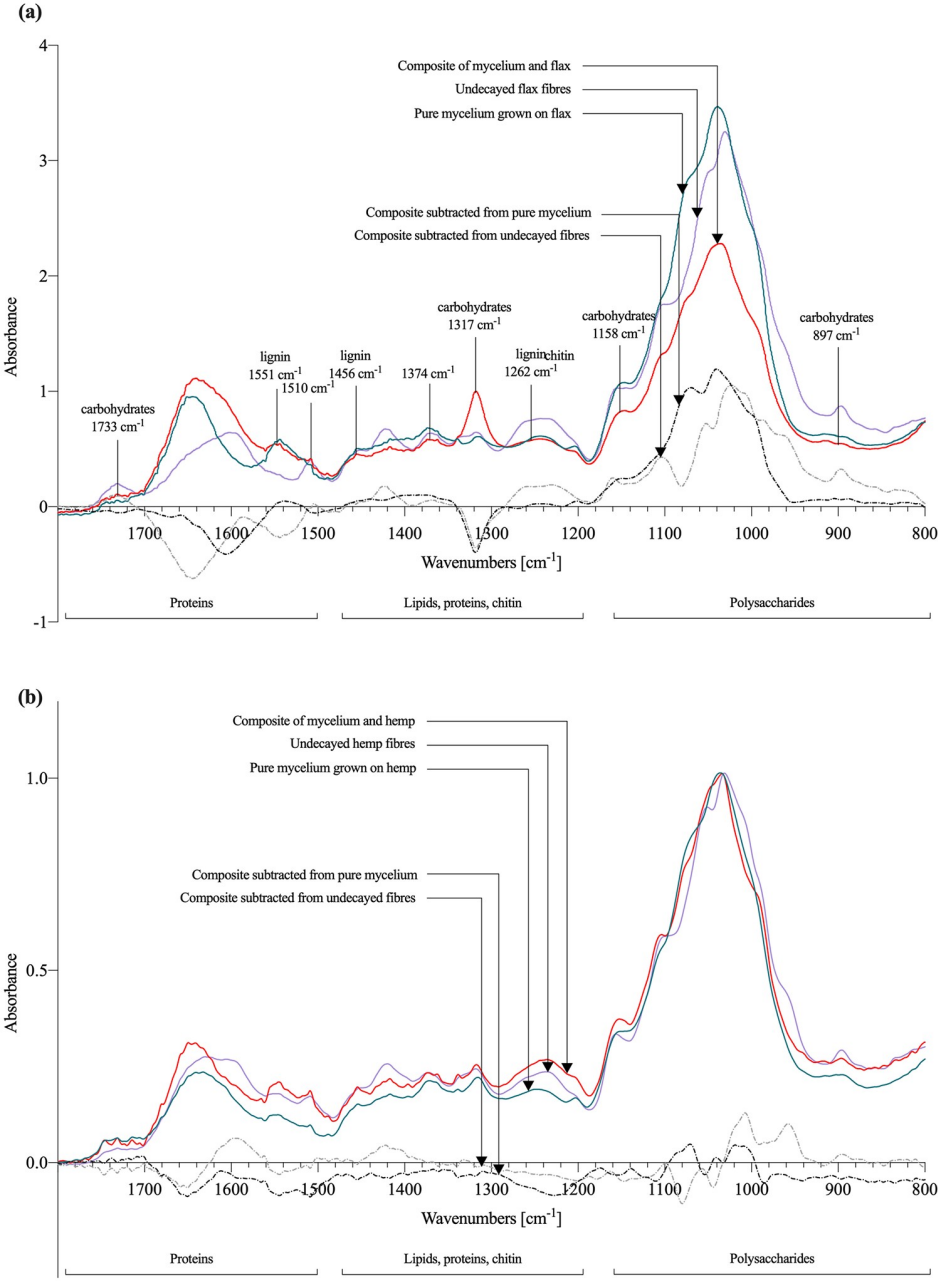
FTIR spectra of flax- and hemp-based mycelium composites. (a) FTIR spectra of undecayed loose flax fibres, loose flax and mycelium composite, pure mycelium grown on flax fibres. (b) FTIR spectra of undecayed loose hemp fibres, loose hemp and mycelium composite, and pure mycelium grown on hemp fibres. The subtracted peaks of dried composite from undecayed fibres (black dash dot line) below the baseline show an appearance of new bands due to the degradation by *T*. *versicolor*, while the subtracted peaks above the baseline show the decrease in intensity. The intensities are around zero when there is no change in the chemical composition of the fibres during the mycelium interaction.

The FTIR spectra of flax-based composites were compared to undecayed fibres, pure mycelium (without fibres) grown on flax, as well as the subtraction peaks in Fig 7(a). The degradation of the flax composites by *T*. *versicolor* led to the small decrease in intensities of carbohydrates at 1733 cm^-1^ (weak), 1158 cm^-1^ (weak), 897.2 cm^-1^ (medium). The increase in carbohydrates was most pronounced at 1317 cm^-1^ (strong). The spectra also revealed an increase in the relative intensities of lignin bands at 1551 cm^-1^ (medium), 1510 cm^-1^ (strong), 1456 cm^-1^ (medium), and 1262 cm^-1^ (weak). The spectra also revealed a small peak at 1374 cm^-1^ (weak) assigned to chitin. New bands for flax composite subtracted from undecayed fibres were created due to the interaction with mycelium (below the baseline) at 1551 cm^-1^ (lignin bands) and 1317 cm^-1^ (cellulose bands). The disappearance of bands (above baseline) for flax composites subtracted from pure mycelium (grey dash dot line) were clear at 1551 cm^-1^ (lignin bands) and 1084 cm^-1^ (cellulose bands).

The FTIR spectra of hemp-based composites were also compared to undecayed fibres, pure mycelium (without fibres) grown on flax, as well as the subtraction peaks in Fig 7(b). The spectra for hemp revealed a decrease of carbohydrates intensities at 1733 cm^-1^ (weak), 1143 cm^-1^ (weak), 1047 cm^-1^ (weak), 896.8 cm^-1^ (medium). The degradation of hemp also resulted in an increase of lignin intensities at 1544 cm^-1^ (strong) and 1231 cm^-1^ (weak), accompanied by a decrease at 1595 cm^-1^, 1508 cm^-1^ (medium) and 1456 cm^-1^ (weak). The appearance and disappearance of bands for the subtracted peaks was less pronounced for hemp fibres compaired to flax. New bands were formed due to the interaction with mycelium at 1076 cm^-1^ and 1010 cm^-1^ (cellulose).

**Fig 8:**
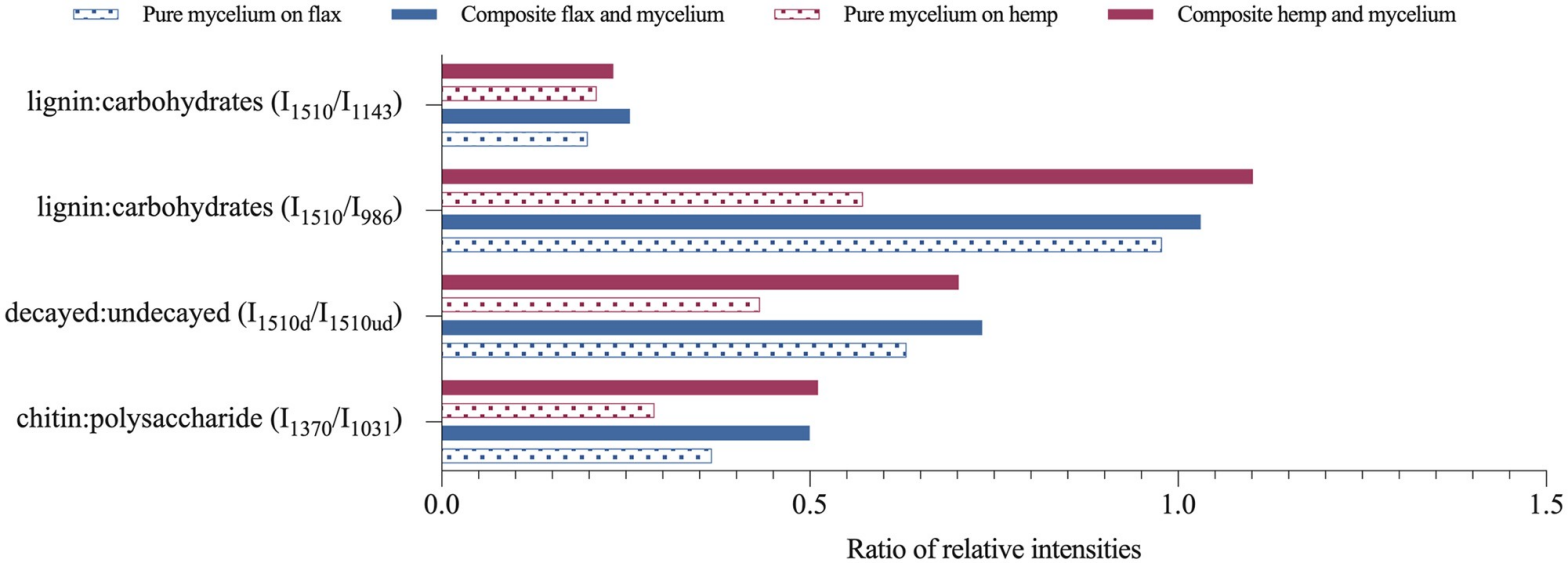
Ratios of the relative intensities. Aromatic skeletal vibration in lignin I_1510_ against undecayed I_1510ud_ fibres, carbohydrate bands I_986_ (assigned to CH-deformation in cellulose) and I_1143_ (assigned to C-O-C vibration in cellulose and hemicellulose), C-H bending mode of chitin I_1370_ and the C-C stretching of polysaccharides I_1031_ of pure mycelium without fibres (pattern), and hemp and flax composite (solid).

Fig 8 compares the type of fibres based on the ratios of the relative intensities. The ratio of lignin:carbohydrate intensities (I_1510_/I_986_ and I_1510_/I_1143_) between hemp and flax dried composites do not differ much. At first sight, this suggests that *T*. *versicolor* decayed lignin, hemicellulose and cellulose in both fibres in the same way. The difference in ratios is more clear for pure mycelium hemp and flax samples, which indicates a lower decay of lignin than cellulose in hemp compared to flax. The decrease in the lignin:carbohydrate ratio, for hemp and flax, is higher for 1143 cm^-1^ band compared to 986 cm^-1^ band, indicating that *T*. *versicolor* has a small preference for hemicellulose over cellulose. Those results are consistent with other studies [17].

The ratio of peak intensity of chitin:polysaccharide (1370 cm^-1^ to 1031 cm^-1^) for hemp and flax was respectively 0,21 and 0,20 in pure mycelium, and 0,23 and 0,26 in the dried composites. Contrary to results reported by Haneef [12], this study didn’t reveal significant differences between both fibres in the synthesis of chitin polymers, resulting from to the use of natural fibres in this study instead of a synthetic culture medium.

The ratio of peak intensities between decayed and undecayed fibres (I_1510d_/I_1510ud_) for pure mycelium, with a ratio of 0,57 for hemp and 0,97 for flax, revealed a higher intensity of lignin band of decayed flax than undecayed flax. The depolymerisation of lignin by *T*. *versicolor* happened to a greater extend in flax than in hemp. Previous studies have suggested that the decomposition of lignin, attributed to the phenol-oxidizing enzymes (laccase and peroxidase) affects adhesion and the composite’s strength. The enzymes promote the cross-linking of lignin-based radicals and increases the stiffness [16,42]. Furthermore, a high amount of cellulose in fibres results in a higher Young’s modulus and tensile strength [42]. Chopped flax fibres indeed showed a higher compressive stiffness (1,18 MPa) compared to chopped hemp (0,77 MPa), but no consistency is found with the stiffness of loose fibres. Generally, the conducted tests reveal that the mechanical performances of the mycelium-based composites depend more on the fibre condition, size, processing, than on the chemical composition.

## Conclusions

The findings of this research provide an important contribution to the field of biological materials, as it presents a comprehensive overview of the production processes, the mechanical, physical and chemical properties of mycelium-based composites. The paper establishes a complete methodology to evaluate the suitability and selection of organic waste-streams for the manufacturing applications. In this study we investigated the requirements to produce mycelium-based composites with different types of lignocellulosic reinforcement fibres combined with a white-rot fungi, *Trametes versicolor*. Together, they formed an interwoven three-dimensional filamentous network binding the feedstock into a lightweight material. This is the first study reporting and correlating the dry density, the Young’s modulus, the compressive stiffness, the stress-strain curves, the thermal conductivity, the water absorption rate of mycelium-based composites by making use of a disclosed protocol with *T*. *versicolor* and five different type of fibres (hemp, flax, flax waste, soft wood, straw) and fibre processing (loose, chopped, dust, pre-compressed and tow). In addition, a Fourier-transform infrared spectroscopy (FTIR) analyse was conducted on undecayed fibres, dried decayed fibres, and pure mycelium to relate the mechanical results of mycelium-composites to the breakdown of lignin, hemicellulose and cellulose by *Trametes versicolor*.

This research showed that the production of mycelium-composites and their mechanical properties are dependent from the fibre types. Poor growth was found for dust flax and dust straw during the first and second growing period. This inconsistency may be due to unavailable nutrients and the absence of air-voids within the composite. Wood- and straw-based composites also resulted in varying growth of *Trametes Versicolor*, consequently not all tests could be conducted with those fibres. A possible explanation for these results may be the difference in mould type for every test. The samples containing flax, hemp and flax-waste resulted in a well-developed composite and reliable results. Nevertheless, internal growth could be increased in further research by optimizing the fabrication procedure with sufficient air circulation in the mould.

Overall, the results (Fig 4a and 4b) suggest that the fibre type (hemp, flax, wood, flax waste) has a smaller influence on the compressive stiffness than the fibre condition (loose, chopped, tow, pre-compressed). The compressive Young’s modulus is higher for all fibre types in chopped condition since the samples were more dense. Samples containing chopped fibres also showed a more coherent and smoother outer layer. As a compressive material, chopped flax-based samples obviously take the lead (1,18 MPa) compared to the chopped hemp-based samples, which might have been affected by the manufacturing process. Previous studies found that the average compressive strength for cotton down woven mat and hemp pitch woven and non-woven mat substrates varied between 0,67 to 1.18 MPa [4]. However, the samples presented in this paper were not produced with mats but with low-graded hurds. Furthermore, the pre-compression of the samples aimed to improve the compressive mechanical properties of mycelium composites. This fabrication method influenced the composite’s mechanical response in a positive way by increasing the Young’s moduli for every tested fibre type and condition. These findings provide additional support for the hypothesis that pre-compressed mycelium-composites enhance the mechanical performance [7].

Although the mechanical properties are not optimal yet, this research has shown that mycelium-composites can fulfil the requirements for thermal insulation foams and have the potential to replace fosile-based composites. The thermal conductivity and water absorption coefficient of the mycelium composites with flax, hemp, and straw have shown an overall good insulation behaviour in all the aspects compared to conventional insulation materials such as rock wool and glass wool (Fig 6). The hemp-based composites have the most interesting properties as an insulation material, with the lowest thermal conductivity 0.0404 [W/ (m*K)] and water absorption coefficient [0.0073 mm/s ^½^]. Compared to previous research, the water absorption is very low thanks to the two-phase fabrication method of the samples and the hydrophobic nature of mycelium. The study demonstrates that the manufacturing process impacts the desirable properties of the composite: the water absorption was much lower compared to previous studies thanks to the growth of a denser outer hydrophobic mycelial layer. However, other properties related to the thermal performances of insulating materials, such as fire resistance [10], aging, acoustics, water vapor diffusion, should be tested in further research.

The ratios of peak intensities of the FTIR spectra (Fig 8) revealed a lower value for hemp pure dried mycelium in lignin over cellulose and hemicellulose compared to flax pure dried mycelium, which indicates a lower decay of lignin than cellulose in hemp compared to flax. The decrease in the lignin:carbohydrate ratio in hemp and flax indicated that *T*. *versicolor* has a small preference for hemicellulose over cellulose. The relative presence of chitin was not influenced by the type of substrate. The increase in ratio of peak intensities between decayed and undecayed flax fibres reveals a higher intensity of lignin band of decayed flax than undecayed flax. The depolymerisation of lignin by *T*. *versicolor* happen to a greater extend in flax than in hemp.

Generally, the conducted tests reveal that the mechanical performances of the mycelium-based composites depend more on the fibre condition, size, processing, than on the chemical composition. The methology used to evaluate the suitability and selection of organic waste-streams proved to be effective for the mycelium-material manufacturing applications. The wide spectrum of options to compose and grow mycelium-composites makes it complex to compare the results with existing literature since every change of variable affects the growth and mechanical behaviour of the composite. Further work is required to improve the growth conditions, to optimize the mechanical properties and to establish a standard fabrication protocol.

## Supporting information

S1 Fig Cross section of the inner growth of undried FL, FWL and FL samples. (a) mycelium chitinous layer, (b) air-void, (c) limited decayed fibre by mycelium.
(TIF)

S2 Fig Growth evolution of a representative selection of the samples HL, FL, FTT, FUT, FD, FWL, WL, SL. (TIF)

S3 Fig Temperature—Time response of the thermal probes for the determination of the thermal conductivity of FC, HC and SC with different currents (A). The mycelium composites had a lower thermal conductivity than water, therefore lower electrical current was used to mark a clear increase of temperature during the test. The temperature changes depending on the applied current during the test. The response of the probe’s temperature is monitored in function of the time. Nonetheless, as expected, the overall results of the calculations did not present large differences. The higher the applied current, the faster the temperature increased, and thus the lower the thermal conductivity. Yet, this fast rise in temperature is not recommended by the standard due to possible errors while readings. Therefore it was more reliable to take the average of the values for thermal conductivity corresponding to a lower applied current.
(TIF)

S4 Fig Stress-strain evolution of mycelium-composites with different types of fibres during uniaxial compression. (TIF)

S1 Table Summary of thermal conductivity, density and moisture content of chopped flax (FC), hemp (HC) and straw (SC) mycelium composites. A different electrical current in the circuit was applied for every sample.
(DOCX)

## References

[1] European Commission, editor. Being wise with waste: the EU’s approach to waste management. Luxembourg: Publ. Off. of the European Union; 2010.

[2] Jones M, Huynh T, Dekiwadia C, Daver F, John S. Mycelium Composites: A Review of Engineering Characteristics and Growth Kinetics. Journal of Bionanoscience. 2017;11: 241–257. doi: 10/gdvp8s

[3] Girometta C, Picco A, Baiguera R, Dondi D, Babbini S, Cartabia M, et al Physico-Mechanical and Thermodynamic Properties of Mycelium-Based Biocomposites: A Review. Sustainability. 2019;11: 281 doi: 10/gfwn9n

[4] Ziegler AR, Bajwa SG, Holt GA, McIntyre G, Bajwa DS. Evaluation of Physico-Mechanical Properties of Mycelium Reinforced Green Biocomposites Made from Cellulosic Fibers. Applied Engineering in Agriculture. 2016;32: 931–938. doi: 10/f9k93x

[5] Sun W, Tajvidi M, Hunt CG, McIntyre G, Gardner DJ. Fully Bio-Based Hybrid Composites Made of Wood, Fungal Mycelium and Cellulose Nanofibrils. Scientific Reports. 2019;9 doi: 10/gfwn93 10.1038/s41598-019-40442-8 PMC6403228 30842558

[6] Islam MR, Tudryn G, Bucinell R, Schadler L, Picu RC. Mechanical behavior of mycelium-based particulate composites. Journal of Materials Science. 2018;53: 16371–16382. doi: 10/gfsztm

[7] Appels FVW, Camere S, Montalti M, Karana E, Jansen KMB, Dijksterhuis J, et al Fabrication factors influencing mechanical, moisture- and water-related properties of mycelium-based composites. Materials & Design. 2018;161: 64–71. doi: 10/gfhz6w

[8] Islam MR, Tudryn G, Bucinell R, Schadler L, Picu RC. Morphology and mechanics of fungal mycelium. Scientific Reports. 2017;7 doi: 10/gchg4x 10.1038/s41598-017-13295-2 PMC5638950 29026133

[9] Jiang L, Walczyk D, McIntyre G, Bucinell R. A New Approach to Manufacturing Biocomposite Sandwich Structures: Mycelium-Based Cores. ASME; 2016 p. V001T02A025. 10.1115/MSEC2016-8864

[10] Jones MP, Lawrie AC, Huynh TT, Morrison PD, Mautner A, Bismarck A, et al Agricultural by-product suitability for the production of chitinous composites and nanofibers utilising Trametes versicolor and Polyporus brumalis mycelial growth. Process Biochemistry. 2019.

[11] Xing Y , Brewer M , El-Gharabawy H , Griffith G , Jones P . Growing and testing mycelium bricks as building insulation materials. IOP Conference Series: Earth and Environmental Science. 2018;121: 022032.

[12] Haneef M, Ceseracciu L, Canale C, Bayer IS, Heredia-Guerrero JA, Athanassiou A. Advanced Materials From Fungal Mycelium: Fabrication and Tuning of Physical Properties. Scientific Reports. 2017;7: 41292 10.1038/srep41292 28117421 PMC5259796

[13] Bennet JW, Wunch KG, Faison BD. Use of fungi in biodegradation Manual of Environmental Microbiology. Second Edition ASM Press Washington, D.C; 2002 pp. 960–971.

[14] ten Have R, Teunissen PJ. Oxidative mechanisms involved in lignin degradation by white-rot fungi. Chem Rev. 2001;101: 3397–3413. 11749405 10.1021/cr000115l

[15] Enoki A, Tanaka H, Fuse G. Degradation of Lignin-Related Compounds, Pure Cellulose, and Wood Components by White-Rot and Brown-Rot Fungi. Holzforschung. 1988;42: 9.

[16] Widsten P, Kandelbauer A. Adhesion improvement of lignocellulosic products by enzymatic pre-treatment. Biotechnology Advances. 2008;26: 379–386. 10.1016/j.biotechadv.2008.04.003 18502077

[17] Pandey KK, Pitman AJ. FTIR studies of the changes in wood chemistry following decay by brown-rot and white-rot fungi. International Biodeterioration & Biodegradation. 2003;52: 151–160. doi: 10/b354tr

[18] Faruk O, Bledzki AK, Fink H-P, Sain M. Biocomposites reinforced with natural fibers: 2000–2010. Progress in Polymer Science. 2012;37: 1552–1596. doi: 10/2wd

[19] Rowell RM. Opportunities for Lignocellulosic Materials and Composites In: Rowell RM, Schultz TP, Narayan R, editors. Emerging Technologies for Materials and Chemicals from Biomass. Washington, DC: American Chemical Society; 1992 pp. 12–27. 10.1021/bk-1992-0476.ch002

[20] Monteiro SN, Lopes FPD, Barbosa AP, Bevitori AB, Silva ILAD, Costa LLD. Natural Lignocellulosic Fibers as Engineering Materials—An Overview. Metall and Mat Trans A. 2011;42: 2963 doi: 10/cpwjnx

[21] ASTM D 5334–00. Standard Test Method for Determination of Thermal Conductivity of Soil and Soft Rock by Thermal Needle Probe Procedure. ASTM Standards; 2000.

[22] NBN EN 12667. Thermal performance of building materials and products—Determination of thermal resistance by means of guarded hot plate and heat flow meter methods—Products of high and medium thermal resistance. NBN; 2001.

[23] NBN EN 1609. Thermal insulating products for building applications—Determination of short term water absorption by partial immersion. 2nd ed. NBN; 2013.

[24] NBN EN ISO 15148. Hygrothermal performance of building materials and products—Determination of water absorption coefficient by partial immersion. 2nd ed. NBN; 2003.

[25] ASTM C 1585–04. Standard Test Method for Measurement of Rate of Absorption of Water by Hydraulic- Cement Concretes. ASTM Standards; 2004.

[26] Yang (Joey) Z, Zhang F, Still B, White M, Amstislavski P. Physical and Mechanical Properties of Fungal Mycelium-Based Biofoam. Journal of Materials in Civil Engineering. 2017;29: 04017030 doi: 10/gc4kq9

[27] ISO 9427:2003. Wood-based panels—Determination of density.

[28] ISO 16979:2003. Wood-based panels—Determination of moisture content.

[29] ASTM D 3501–94. Standard Test Methods for Wood-Based Structural Panels in Compression. ASTM Standards; 1994.

[30] Jones M, Huynh T, John S. Inherent species characteristic influence and growth performance assessment for mycelium composite applications. AML. 2018;9: 71–80. doi: 10/gf3bg8

[31] Lelivelt RJJ, Lindner G, Teuffel PM, Lamers HM. The production process and compressive strength of Mycelium-based materials. 2015;

[32] Imhof B, Gruber P. Built to Grow-Blending architecture and biology. Birkhäuser; 2015.

[33] Moser FJ, Wormit A, Reimer JJ, Jacobs G, Trautz M, Hillringhaus F, et al. Fungal mycelium as a building material [Internet]. Fachgruppe Biologie, Lehrstuhl für Botanik und Institut für Biologie I (Botanik), Lehrstuhl und Institut für Allgemeine Konstruktionstechnik des Maschinenbaus, Lehrstuhl für Tragkonstruktionen; 2017. Report No.: RWTH-2017-08964. http://publications.rwth-aachen.de/record/706992

[34] Rockwool. Product data sheet: Rockwool Energysaver [Internet]. 2011. https://static.rockwool.com/globalassets/rockwool-uk/downloads/datasheets/walls/energysaver-data-sheet.pdf

[35] Papadopoulos AM. State of the art in thermal insulation materials and aims for future developments. Energy and Buildings. 2005;37: 77–86. doi: 10/fgpdrz

[36] Asdrubali F, D’Alessandro F, Schiavoni S. A review of unconventional sustainable building insulation materials. Sustainable Materials and Technologies. 2015;4: 1–17. doi: 10/gfhwng

[37] Appels F, Dijksterhuis J, Lukasiewicz CE, Jansen KMB, Wösten HAB, Krijgsheld P. Hydrophobin gene deletion and environmental growth conditions impact mechanical properties of mycelium by affecting the density of the material. Scientific Reports. 2018;8 doi: 10/gc8d9m 10.1038/s41598-018-23171-2 PMC5856774 29549308

[38] Wessels JGH. Hydrophobins: Proteins that Change the Nature of the Fungal Surface In: Poole RK, editor. Advances in Microbial Physiology. Academic Press; 1996 pp. 1–45. 10.1016/S0065-2911(08)60154-X 8922117

[39] Mohebby B. Attenuated total reflection infrared spectroscopy of white-rot decayed beech wood. International Biodeterioration & Biodegradation. 2005;55: 247–251. doi: 10/dv4895

[40] Schwanningera M, Rodriguesc JC, Pereirac H, Hinterstoisserb B. Effects of short-time vibratory ball milling on the shape of FT-IR spectra of wood and cellulose. Vibrational Spectroscopy. 2004;36: 23–40. 10.1016/j.vibspec.2004.02.003

[41] Popescu C-M, Popescu M-C, Vasile C. Structural changes in biodegraded lime wood. Carbohydrate Polymers. 2010;79: 362–372. doi: 10/d6zqqf

[42] John M, Thomas S. Biofibres and biocomposites. Carbohydrate Polymers. 2008;71: 343–364. doi: 10/cvpmrh

