# Supplementary figures and images for: Mechanical, physical and chemical characterisation of mycelium-based composites with different types of lignocellulosic substrates
Source: PLoS One. 2019 Jul 22;14(7):e0213954. doi: 10.1371/journal.pone.0213954 (PMC6645453; doi:10.1371/journal.pone.0213954)

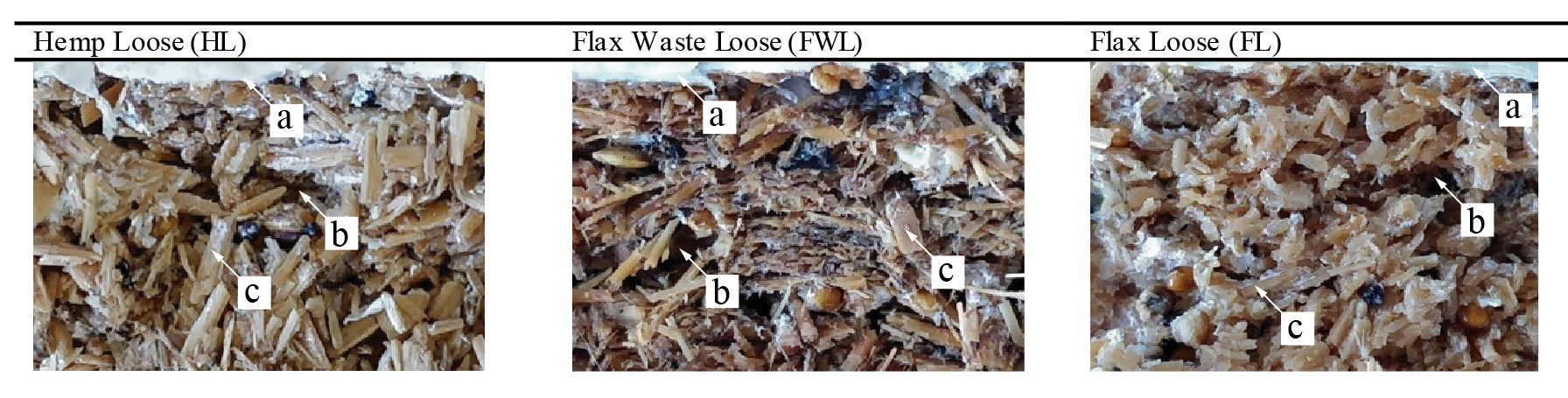

Supplement: S1 Fig — (a) mycelium chitinous layer, (b) air-void, (c) limited decayed fibre by mycelium. (TIF) [file pone.0213954.s001.tif]

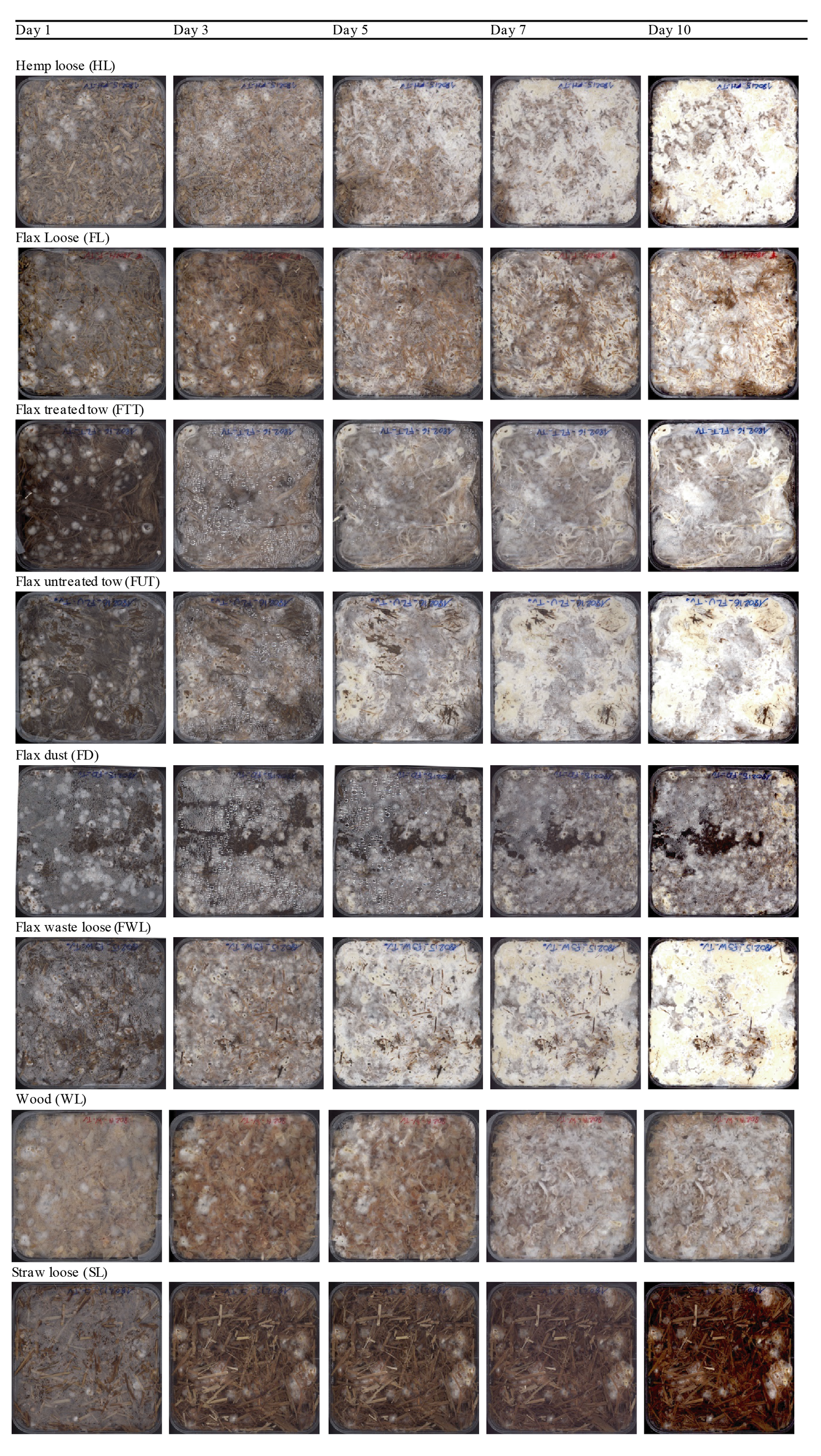

Supplement: S2 Fig — (TIF) [file pone.0213954.s002.tif]

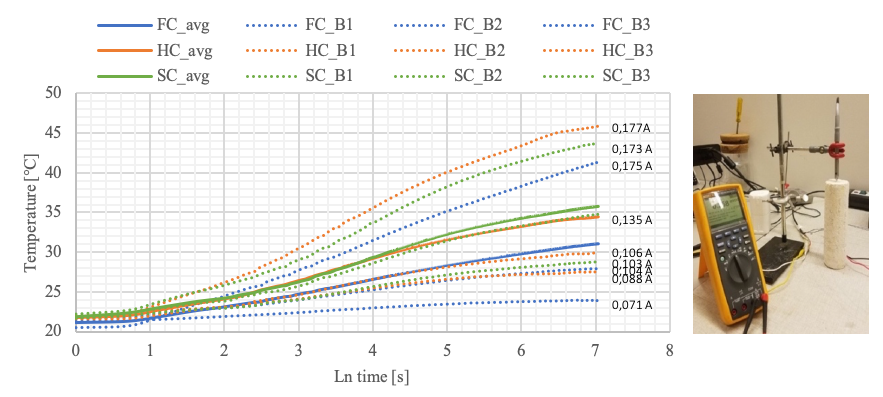

Supplement: S3 Fig — The mycelium composites had a lower thermal conductivity than water, therefore lower electrical current was used to mark a clear increase of temperature during the test. The temperature changes depending on the applied current during the test. The response of the probe’s temperature is monitored in function of the time. Nonetheless, as expected, the overall results of the calculations did not present large differences. The higher the applied current, the faster the temperature increased, and thus the lower the thermal conductivity. Yet, this fast rise in temperature is not recommended by the standard due to possible errors while readings. Therefore it was more reliable to take the average of the values for thermal conductivity corresponding to a lower applied current. (TIF) [file pone.0213954.s003.tif]

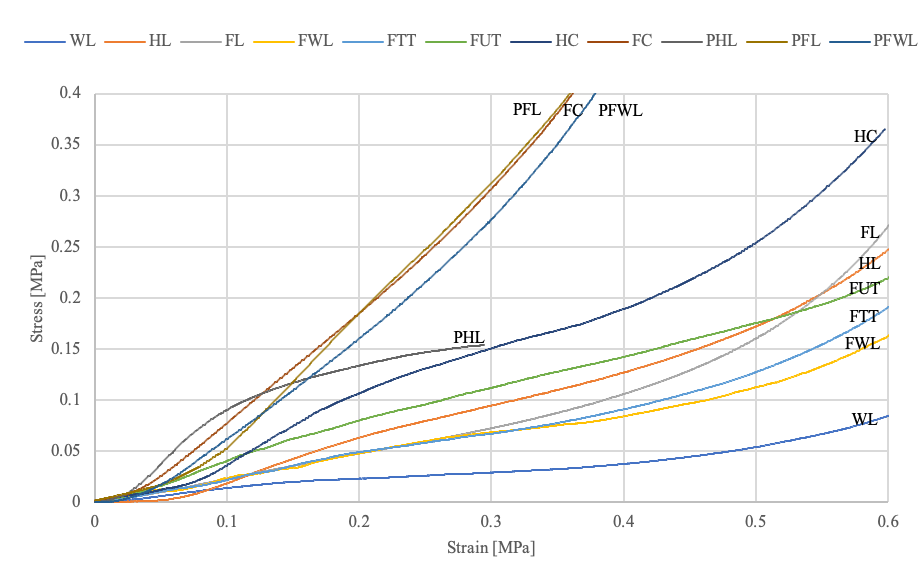

Supplement: S4 Fig — (TIF) [file pone.0213954.s004.tif]
