# Supplementary material for: Mechanical, physical and chemical characterisation of mycelium-based composites with different types of lignocellulosic substrates
Source: PLoS One. 2019 Jul 22;14(7):e0213954. doi: 10.1371/journal.pone.0213954 (PMC6645453; doi:10.1371/journal.pone.0213954)
Supplement: S1 Table — A different electrical current in the circuit was applied for every sample. (DOCX) [file pone.0213954.s005.docx]

**S1 Table: Summary of thermal conductivity, density and moisture content of chopped flax (FC), hemp (HC) and straw (SC) mycelium composites.**

| Fibre type | Current in TNP  [A] | Density [Kg/m^3^] | Thermal conductivity (λ)  [W/ (m*K)] | Average (λ)  [W/ (m*K)] | Moisture content [%] |
| --- | --- | --- | --- | --- | --- |
| FC_B1 | 0.104 | 134.71 | 0.0582 | 0.0578 | 9,5% |
| FC_B2 | 0.071 |  | 0.0603 |  |  |
| FC_B3 | 0.175 |  | 0.0550 |  |  |
| HC_B1 | 0.106 | 98.92 | 0.0409 | 0.0404 | 7,4% |
| HC_B2 | 0.177 |  | 0.0417 |  |  |
| HC_B3 | 0.088 |  | 0.0386 |  |  |
| SC_B1 | 0.103 | 94.39 | 0.0420 | 0.0419 | 13% |
| SC_B2 | 0.135 |  | 0.0421 |  |  |
| SC_B3 | 0.173 |  | 0.0417 |  |  |
